# Physical and mental fatigue in shift work and mitigation strategies: an integrative review

**DOI:** 10.47626/1679-4435-2024-1267

**Published:** 2025-01-13

**Authors:** Amanda Sorce Moreira, Sergio Roberto de Lucca

**Affiliations:** 1 Public Health, Universidade Estadual de Campinas, Campinas, SP, Brazil

**Keywords:** fatigue, shift work schedule, work, occupational risks., fadiga, jornada de trabalho em turnos, trabalho, riscos ocupacionais.

## Abstract

The new configurations of society have fragmented working hours into shifts, resulting in greater fatigue which affects worker health. Our aim was to identify the relationship between physical and mental fatigue and shift work and the main strategies for mitigating these effects. This review study was conducted between March and May 2023 using the *Biblioteca Virtual em Saúde* (Virtual Health Library). Twenty seven of the 1,176 identified articles were selected, with health professionals (doctors and nursing professionals), drivers, and aircraft pilots being the most studied populations. The following strategies for mitigating fatigue in shift work stood out: work schedule adjustments, interand intra-workday rest associated with phototherapy, monitoring and evaluating early signs of fatigue, supervised prescription of stimulants (such as caffeine) and sedatives, use of monitoring equipment, and staff education and training.

## Abstract (pt)

As novas configurações da sociedade fragmentaram as jornadas de trabalho em turnos, resultando em maior fadiga e impactando a saúde dos trabalhadores. O objetivo deste trabalho foi identificar a relação entre a fadiga física e mental e o trabalho em turnos, além das principais estratégias utilizadas para mitigar esses efeitos. Foi realizado um estudo de revisão bibliográfica integrativa no período de março a maio de 2023, na Biblioteca Virtual em Saúde. Foram selecionados 27 artigos dos 1.176 identificados, sendo os profissionais de saúde (médicos e enfermeiros), motoristas e pilotos de aeronaves as populações mais estudadas. Entre as estratégias para mitigar a fadiga no trabalho em turnos, destacam-se: ajustes na escala de trabalho; descansos inter e intrajornada associados ao uso de óculos de fototerapia; monitoramento e avaliação de sinais precoces de fadiga; prescrição supervisionada de substâncias estimulantes (por exemplo, cafeína) ou indutoras do sono; uso de equipamento de monitoramento; e educação e treinamento da equipe.

## INTRODUCTION

The contemporary world of work demands ever greater productivity to meet the demands of consumer society, which has led many services and sectors of industry to operate 24 hours a day. The shift work system has made it possible to continue this process without interruption, but requires workers to adapt to company-determined schedules.^1^

There are basically two ways to organize this type of work: fixed shifts and rotating shifts, which can be short or long, with variable rest periods. The shift schedule is also variable (morning, afternoon, or night), as is the number of work days and days off between shifts. Thus, shifts can vary between different combinations of days on and off (6 × 1, 6 × 2, 5 × 2, 4 × 3, 12 × 36, etc.) or, in offshore contexts, shifts can be 15 × 15, for example.^1^

Long, night, and rotating shifts can substantially harm worker health because they alter circadian rhythm regulation due to insufficient rest to reestablish homeostasis and balance between sleep and wakefulness processes. This can trigger malaise, fatigue, mood changes, drowsiness, depressive symptoms, irritability, anxiety and muscle pain.^2^-^4^ These effects can pose safety risks to both workers and service users, especially in the health care and transportation sectors.^1^,^2^

Errors often arise from fatigue due to the depletion of individual resources through repetitive activities, long working hours, physically and mentally demanding tasks, irregular shift work, and night shifts. Fatigue, which can persist for long periods, is not relieved by sleep and rest alone, and it substantially impacts health, safety, and work performance.^5^

Fatigue is clinically manifested by a subjective feeling of tiredness or exhaustion that originates from dysfunction in the neuroendocrine-adrenal regulatory system due to stimulus overload (physical and/or cognitive workload) and an inability to reestablish balance. Due to various triggering factors, fatigue generates a series of physiological changes that result in glandular and muscular changes due to physical overload, in addition to mental fatigue due to excess information. Both forms can evolve into chronic fatigue through continuous exposure to high levels of stress.^6^

Physiologically, fatigue results from dysfunction between dopamine and cortisol.^6^ Some manifestations of fatigue, such as irritability, discouragement, and lack of desire to go to work, have been associated with high cortisol levels, as well as immunoglobulin type A, which, in addition to being related to these manifestations, is associated with depressive symptoms in fatigued workers.^7^ In addition to fatigue, the inability to work shifts can increase blood pressure, mean insulin levels, and some inflammatory biomarkers, such as tumor necrosis factor alpha, leptin, and interleukin-6.^7^ Workers with a morning or evening chronotype tend to experience greater fatigue and poorer sleep quality during their shifts. Thus, these biomarkers may be an important early indicator of shift work-related fatigue.^6^-^9^

Because of the high prevalence of shift work and its impact on health and quality of life through fatigue, this review aimed to identify the relationship between physical and mental fatigue and shift work and the main strategies used to mitigate their effects.

## METHODS

This integrative literature review was prepared according to the PICO strategy:^10^ Population - workers in general, Investigated condition - manifestations of fatigue, and Comparison condition - shift work. The study question was: “What evidence is there that shift work contributes to symptoms of physical and mental fatigue and what strategies have been applied to prevent or mitigate its effects?”

The Brazilian Ministry of Health’s *Biblioteca Virtual de Saúde* (Virtual Health Library), which includes databases such as MEDLINE/PubMed, Latin American and Caribbean Health Sciences Literature (LILACS), and other sources of scientific content, was searched between March and May 2023.

Due to the interconnectivity between fatigue’s physical and mental manifestations, most publications only mention the term “fatigue”. Thus, the keywords “*Fadiga*”, “Fatigue”, “*Jornada de trabalho em turnos*” and “Shift Work Schedule”, combined with the Boolean operator “AND”, were used to search for articles. To expand the search, the uncontrolled keywords “*Turno*” and “Shift” were also used in combination with “*Fadiga*” and “Fatigue”.

Because the study’s aim was to identify more recent publications to highlight updates on fatigue and prevention strategies, only open access full texts published between 2018 and 2023 in English, Spanish, or Portuguese were included.

**Figure 1:**
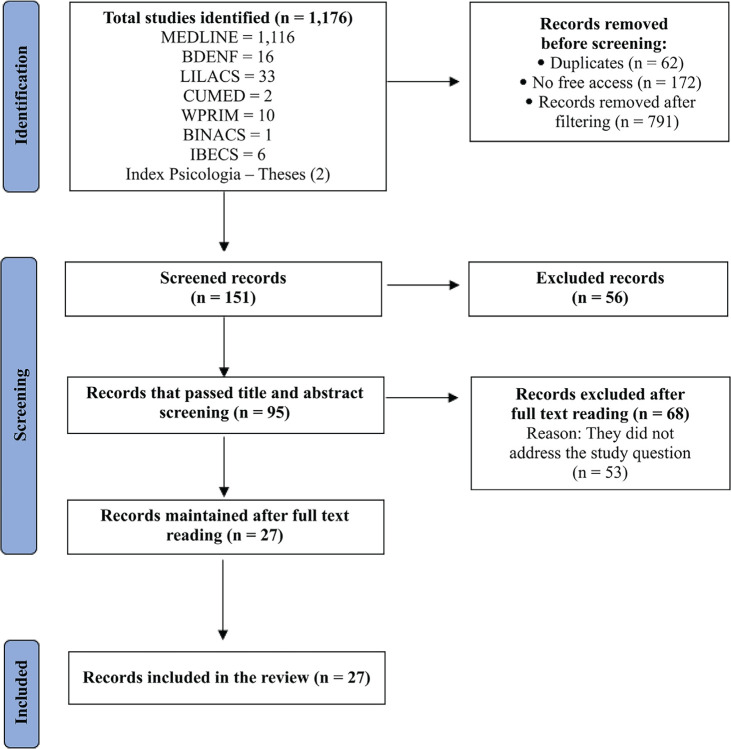
Article selection flowchart according to the Preferred Reporting Items for Systematic Reviews and Meta-Analyses. BDENF = *Base de Dados de Enfermagem* (Nursing Database); LILACS = *Literatura Latino-Americana e do Caribe em Ciências da Saúde* (Latin American and Caribbean Health Sciences Literature); CUMED = *Base de Datos de Bibliografía Médica Cubana* (Cuban Medical Bibliography Database); WPRIM = Western Pacific Regional Index Medicus; BINACS = *Base de Dados de Informação em Ciências da Saúde* (Health Sciences Information Database); IBECS = *Índice Bibliográfico Español en Ciencias de la Salud* (Spanish Bibliographic Index in Health Sciences).

The selection and analysis processes were performed by two independent researchers. Data from all eligible studies were entered into an Excel spreadsheet and assessed according to the Preferred Reporting Items for Systematic Reviews and Meta-Analyses methodology, as described in Figure 1.

## RESULTS

A total of 27 articles that addressed the study question were identified. They were published mainly in English (26) and in 2019 (8). The most frequently studied populations were health care professionals (doctors and nurses), drivers, and aircraft pilots. The studies aimed to quantify fatigue in workers, identify fatigue-related risk factors and propose measures to mitigate the effects of fatigue in shift work (Table 1).

**Table 1:** Description of the selected articles

Title	Authors	Year of publication	Objectives	Main results
Working Time Society consensus statements: a multi-level approach to managing occupational sleep-related fatigue	Wong et al.^1^	2019	To compare and contrast the diversity and evolution of today’s regulatory practice landscape and help organizations and workers find solutions to fatigue.	Approaches to mitigating shift work risks are critical to protecting the health and safety of workers and should be based on a prescribed set of rules, risk management principles, or a combination of both.
Doctors’ extended shifts as risk to practitioner and patient: South Africa as a case study	Kotze et al.^2^	2020	To explore contextual factors that could influence attempts to mitigate fatigue.	Working at night or in shifts always involves health risks. Long or consecutive shifts must be regulated.
Shift work adaptation among police officers: the BCOPS study	Nevels et al.^8^	2021	To examine adaptation to shift work among police officers and potential differences in biomarkers of disease between maladapted and adapted shift workers.	Elevated leptin and insulin may be related to day shift work or to workers who have not adapted to the shift. Workers who are poorly adapted to shift work tend to have lower social support and higher levels of stress, depression, and fatigue. Several lifestyle or personality characteristics have been associated with adaptation to shift work, including diet (vegetable intake/day), strength, lower levels of neuroticism and family conflict. Workers who are poorly adapted to shift work may have higher levels of interleukin-6, leptin, tumor necrosis factor alpha, and insulin.
Fatigue management in health care: it is a risky business	Dawson & Thomas^11^	2019	To describe the organization of working time, fatigue and self-reported well-being by physicians.	Working hours should be adjusted to the group of workers. Fatigue-related risks are highly variable and reflect other factors, such as work shift, type of work, workload, management, lack of social support, etc., and should be considered when assessing the level of fatigue. Assessing sleep and wakefulness during shifts is the most effective way to quantify and control fatigue-related risks. Analyzing and redesigning workflows in which fatigue-related errors have occurred can significantly reduce the occurrence of new events.
The effects of individual characteristics of the naval personnel on sleepiness and stress during two different watchkeeping schedules	Myllylä et al.^12^	2022	To investigate the individual characteristics of U.S. Navy personnel associated with levels of sleepiness, fatigue, and stress response during shift work and irregular hours in their work environment.	Older age and longer service years are associated with greater sleepiness, fatigue, and stress during shift and rotational work. Regular physical activity and a higher level of physical fitness appear to be more clearly associated with less sleepiness, fatigue, and stress.
Improving fatigue risk management in health care: a systematic scoping review of sleep-related/fatigue-management interventions for nurses and midwives	Querstret et al.^13^	2020	To identify interventions related to sleep and fatigue management, to systematically review all empirical evaluations of fatigue management interventions, to identify the methods used by researchers to measure the effectiveness of these interventions, and to develop a map of interventions and measures in this domain for nurses and midwives.	Night shifts, long shifts, and few hours of rest between shifts worsen sleep quality and increase fatigue. Interventions based on light exposure and attenuation and intra-shift naps show promise in reducing fatigue.
A field investigation of the relationship between rotating shifts, sleep, mental health and physical activity of Australian paramedics	Khan et al.^14^	2021	To investigate the relationship between rotating shifts, sleep, mood, stress, fatigue, sleepiness, energy expenditure and physical activity levels among Australian paramedics.	Fatigue levels are highest on the last day of night work, peaking on the first day off. Sleep restriction (averaging 4 hours per day) can increase fatigue. Workers with greater physical demands on the night shift experience greater fatigue and physical exhaustion. Rotational work is associated with increased sleepiness, stress, and fatigue, but not with mood.
An exploration of shift work, fatigue, and gender among police officers: the BCOPS study	Violanti et al.^15^	2018	To examine the association between shift work and tiredness, a symptom of fatigue, among urban police officers and to assess whether this association varies by gender.	Fixed shift work was not associated with fatigue. Male officers working the afternoon shift were more likely to feel more tired than those working the morning and night shifts, regardless of sleep hours or work demands.
Condicionantes de calidad de los cuidados enfermeros durante el turno de noche	Salas Marco et al.^16^	2022	To identify conditions related to the quality of nursing care during night shifts in hospitals.	The quality of care during the night shift is lower than that observed during other shifts. The main effects of night work on nurses are disruptions in the circadian cycle, sleep disorders, increased health problems, and social and domestic repercussions, which can lead to emotional exhaustion, stress, anxiety, fatigue, burnout, and signs of depression. Fatigue affects the cognitive process, reducing stress tolerance, the ability to respond to stimuli, and critical thinking. Rotating shifts and overtime were more related to fatigue. Fatigue is related to deterioration in the quality of care, lower satisfaction, and higher absenteeism.
Comparing the acute effects of shiftwork on mothers and fathers	Tucker et al.^17^	2021	To analyze whether the combination of shift work and childcare affects sleep, fatigue and work-family conflict in women more than in men.	Shift work, female sex, and the presence of young children at home were associated with negative effects on sleep, fatigue, and work-family conflict.
Interactions between home, work, and sleep among firefighters	Watkins et al.^18^	2021	To assess family dynamics and how firefighters prioritize sleep and recovery at home, based on family relationships and work shifts.	Having a partner may increase compassion fatigue due to feelings of trying to improve or maintain the relationship at the expense of insufficient recovery from sleep debt. Individual interventions, such as promoting physical, mental, and social health, and organizational interventions, such as implementing sleep hygiene practices (using blackout curtains in bedrooms, establishing a rest room, providing comfortable sleeping mats, controlling noise, lighting, and outdoor temperature) and adjusting work shift schedules, may improve recovery at home.
Working conditions and fatigue in Japanese shift work nurses: a cross-sectional survey	Kida & Takemura^19^	2022	To identify working conditions associated with fatigue based on shift patterns and the number of holidays and to determine the threshold of associated conditions.	Rotations between two shifts and long working hours on both day and night shifts were associated with high levels of fatigue. Rotations between three shifts, consecutive days of working a night shift at midnight, and returning to the day shift at midnight were associated with high fatigue. Each shift rotation pattern requires different strategies to mitigate the effects of fatigue.
A study on fatigue management of aviation maintenance mechanics-focusing on shift workers	Kim & Choi^20^	2020	To develop a plan to minimize fatigue in aircraft maintenance technicians by examining factors that may affect them during day and night work.	Fatigue increases gradually with the work schedule (morning < afternoon < night). The longer the daily workday, the greater the fatigue. Long shifts increase the chance of errors and the risk of accidents due to fatigue. Physically demanding tasks should not precede monotonous tasks or tasks that require concentration. The presence of a professional to supervise shift work reduces errors due to physical fatigue. Shift work, especially night work, becomes more challenging as the worker ages.
Guiding principles for determining work shift duration and addressing the effects of work shift duration on performance, safety, and health: guidance from the American Academy of Sleep Medicine and the Sleep Research Society	Gurubhagavatula et al.^21^	2021	To propose guiding principles to assist in the development of a decision-making process on shift lengths, effectively balancing the need to meet operational demands with the management of fatigue-related risks.	Currently, shift lengths are based on the balance between workplace productivity and the risks associated with physical fatigue, without taking into account the impact on mental fatigue and the risks associated with performance, safety and health. Shift length assessments should be context-specific and based on a trade-off assessment that incorporates a thorough assessment of needs and risks. A key question when determining shift lengths is whether the risks of continuing to work outweigh the risks of not working.
Fatigue risk management: the impact of anesthesiology residents’ work schedules on job performance and a review of potential countermeasures	Wong et al.^22^	2018	To review current Accreditation Council for Graduate Medical Education guidelines on resident work hours, to examine how anesthesiologists’ work schedules may affect professional performance, to discuss the ramifications of nighttime and extended duty hours on patient safety and resident well-being, and to propose and describe countermeasures to mitigate the effects of fatigue.	Acute and chronic sleep deprivation, sleep inertia, and circadian rhythm misalignment can induce significant deficits in performance. Long work hours and night shifts can increase the effects of fatigue, which increases susceptibility to medical errors and vehicle crashes. Adjusting work schedules, taking naps, taking microbreaks, using stimulants, and exposing workers to bright and/or blue light can be viable measures to reduce fatigue. Napping, using caffeine, and exposing workers to light represent the most practical combination to increase alertness and improve performance during periods of wakefulness and transition to the night shift.
How to schedule night shift work in order to reduce health and safety risks	Garde et al.^23^	2020	To provide scientifically-based recommendations on night shift schedules, including consecutive shifts, shift breaks, and shift lengths, to minimize health and safety risks.	Sleepiness is most pronounced on the first day of night work and is an unavoidable effect. Fatigue risk management includes measures to combat sleepiness beyond shift scheduling, such as the use of technologies that assess previous sleep data and fatigue detection technologies. The use of bright light, melatonin, naps, and stimulants are ways to improve adaptation to night shift work.
Should public safety shift workers be allowed to nap while on duty?	Patterson et al.^24^	2020	To define the intraday nap, summarize the arguments for and against it, and outline potential applications of this important fatigue mitigation strategy in emergency medical services workers.	Intra-shift napping is an effective measure to mitigate fatigue during shift work. Taking naps between 1:00 p.m. and 5:00 p.m. or between 1:00 a.m. and 5:00 a.m. is most effective against fatigue during shift work. Naps lasting 50 minutes between midnight and 4:00 a.m. have been shown to benefit reaction time, performance, lapses, and memory.
The effects of a 120-minute nap on sleepiness, fatigue, and performance during 16-hour night shifts: a pilot study	Oriyama et al.^25^	2019	To investigate sleepiness, fatigue, and performance after a 120-minute nap during 16-hour night shifts based on subjective and objective assessments.	Taking 120-minute naps between midnight and 4:00 a.m. is effective in reducing fatigue, drowsiness and the risk of error when working the night shift.
Working Time Society consensus statements: regulatory approaches to reduce risks associated with shift work-a global comparison	Gärtner et al.^26^	2019	To provide guidance on how to manage fatigue associated with non-standard work schedules and to ensure the health and safety of workers.	Prescribed approaches to reducing the risks of fatigue associated with shift work should include maximum work limits for a single shift and for periods of 1 to 4 weeks, as well as minimum rest limits for time off during a shift and for time off between consecutive shifts. Maximum work limits for a single shift and minimum rest limits for time off between consecutive shifts should vary according to the time of day at which work and rest occur.
Rotating shifts negatively impacts health and wellness among intensive care nurses	Imes & Chasens^27^	2019	To describe correlations between patient-reported risk factors in the Health and Well-Being Outcomes Measurement Information System and to examine differences in self-reported measures among participants after night shift work compared with day shift work.	Shift rotation negatively affects nurses’ health and well-being. The relationship between impairment and sleep was greater on the night shift and is highly related to greater fatigue, increased emotional distress (anger) and worse cognitive abilities (memory and concentration).
Can nurses’ shift work jeopardize the patient safety? A systematic review	Di Muzio et al.^28^	2019	To analyze the correlation between clinical risk management, the occurrence of medication errors and the effects of shift work on nurses.	Fatigue is the main risk factor for medication errors. Shifts > 12 h/day and 40 h/week cause greater fatigue and lead to an increase in the rate of adverse events and medication errors. Working three or more consecutive shifts is more related to errors. Night nurses feel more fatigue and make more errors than day nurses. A reduced number of professionals on the schedule and the type of care provided increase fatigue and the occurrence of errors.
Fatigue analysis of high dump truck operators in Indonesia’s coal mining industry: a case study	Mulyati et al.^29^	2020	To analyze fatigue conditions and deepen the effect of work shifts on drivers of high-capacity dump trucks in a coal mining operation.	Truck drivers who work the night shift experience more fatigue than those who work the day shift, leading to reduced concentration due to staying awake at night and sleeping during the day. The level of fatigue is related to errors and the high weekly workload. Dividing the day (24 hours) into three work shifts helps reduce fatigue. Providing music that helps restore energy, in addition to providing adequate rest between shifts, helps reduce fatigue.
Working Time Society consensus statements: prescriptive rule sets and risk management-based approaches for the management of fatigue-related risk in working time arrangements	Honn et al.^30^	2019	To provide examples of control measures along each level of the fatigue risk trajectory.	Sleep-related fatigue associated with shift work is a complex hazard that results from a variety of work-related and non-work-related sources. Fatigue management should consider: providing adequate opportunities for rest; ensuring good quality sleep; identifying and managing fatigue-related behaviors and symptoms; and recognizing fatigue as a cause of adverse events.
Improved sleep quality and work performance among shift workers consuming a “foods with function claims” containing asparagus extract	Sakai et al.^31^	2021	To verify whether foods with functional property claims containing asparagus extract effectively improve sleep quality and performance in shift workers.	Consuming foods containing asparagus extract helps improve sleep quality, reduce feelings of fatigue and improve shift work performance.
A quasi-experimental study into the effects of naps and therapy glasses on fatigue and well-being	van Woerkom^32^	2021	To investigate the effects of napping and the use of therapy glasses on fatigue and well-being at the end of a night shift.	Taking naps during night work reduces fatigue. The use of phototherapy glasses (blue lens, Propeaq®) also helps reduce fatigue during night work.
Sleep and transportation safety: role of the employer	Rainey et al.^33^	2019	To disseminate strategies to mitigate fatigue in drivers who work shifts.	The main preventive measures to reduce the risk of fatigue in drivers include working time management; educational programs on fatigue and its effects; non-punitive organizational strategies in cases of fatigue reporting; using technologies to identify fatigue in vehicles; and assessing fatigue-related risk factors among drivers.
Effect of fatigue training on safety, fatigue, and sleep in emergency medical services personnel and other shift workers: a systematic review and meta-analysis	Barger et al.^34^	2018	To critically review and synthesize existing literature on the impact of fatigue training on outcomes in emergency medical professionals and similar groups of shift workers.	Fatigue training has been shown to be beneficial for shift workers, increasing care quality, patient safety, acute fatigue, professional development, and quality of life, and to reduce burnout and stress.

## DISCUSSION

Risk factors for shift work-related fatigue are highly variable, including shift type, shift rotation, work type and demand, managerial profile, and lack of social support.^11^ In addition to these factors, individual characteristics, such as age, sex, family issues, time in the current job position, and lifestyle habits, can increase the risk of fatigue and burnout symptoms.

Age and time in the current job position can increase a worker’s predisposition to fatigue, given the difficulty of adaptation and greater exposure to irregular hours.^12^,^13^ Although physical activity is generally associated with lower fatigue, drowsiness, and stress,^12^ night shift workers may experience greater exhaustion during physical activity when performed at times counter to their biological clock.^14^

Perceived fatigue differs between the sexes. Women tend to report greater fatigue at the end of morning shifts, while men report twice as much fatigue at the end of afternoon shifts.^15^ Furthermore, women who work shifts and have young children experience more sleep problems and consume more psychotropic medications than men.^16^,^17^

The double shift (i.e., paid employment and housework) that many women face increases compassion fatigue, a syndrome that includes physical, psychological and social exhaustion resulting from the attempt to maintain or improve family relationships at the expense of insufficient recovery. This increases family conflicts and the risk of accidents both at home and in the workplace.^18^

The relationship between shift work and fatigue is clear, and finding strategies to mitigate its effects has been a challenge, since each pattern of shift rotation demands different strategies to alleviate fatigue.^19^ Fatigue increases in the second and third (night) shifts, with the night shift being the most damaging type. When combined with long work hours, overtime, and high demand, night work causes a marked reduction in alertness and contributes to mental illness, increasing the need for inter-shift recovery.^1^,^13^,^19^

Shift lengths have been based on a trade-off between workplace productivity and the risks associated with physical fatigue, often without considering the impact on mental fatigue and the associated risk to performance, safety, and health. Thus, shifts should be analyzed in a context-specific way that is based on a trade-off assessment that incorporates a thorough investigation of needs and risks.^20^

The risk of fatigue increases exponentially with the number of hours worked.^1^ The work day should be based on an understanding of work hours, shift length, rest days between shifts, and the number of successive work days, resulting in schedules that provide adequate time for sleep and recovery.^1^,^21^

The strategy of extending work hours and reducing the number of consecutive days worked (e.g. 12-hour shifts over 4 days) does not reduce the risk of fatigue and it negatively impacts alertness, especially during night shifts. Therefore, the most effective intervention against fatigue is to limit the total number of working hours to a maximum of 8 hours per day for 5 consecutive days.^1^,^21^,^22^

Body temperature regulation and adaptation to the work schedule and shift type are the main factors in performance, safety, and health. Thus, shift rotations must follow the chronobiological principles of occupational health, i.e. a morning, afternoon, and evening sequence, rather than the opposite, since the greatest drop in body temperature occurs at night, causing drowsiness and changes in reasoning ability.^21^,^22^

Night workers tend to be more fatigued after the first day of work. Thus, to establish a good shift schedule, rest breaks during and between shifts must be considered.^20^-^23^ Short frequent breaks of up to 30 minutes between midnight and 6:00 a.m. are recommended, since they protect health, increase performance, and reduce errors. These breaks help maintain aspects such as weight control, blood pressure, and circadian hormone balance.^19^,^23^-^25^ For day shift workers, breaks can be taken between 1:00 p.m. and 5:00 p.m.,^24^ while, for professional drivers, 45 minutes of rest are needed after a maximum driving time of 4.5 hours.^26^

Taking naps during work breaks is highly recommended, especially for night shift workers and those who perform activities that require quick responses.^19^,^23^-^25^ However, in night shift workers, these naps should not exceed 120 minutes and should not occur before midnight, since they can increase fatigue due to the reduction in body temperature that occurs after long naps.^25^

For adequate recovery between work shifts, at least 11 hours of rest are necessary, given that most workers require at least 7 hours of sleep per night and must dedicate part of their free time to a range of activities, such as household chores, childcare or even a second job.^1^,^23^ For those who work long or successive shifts, inter-shift rest should ideally include two nights of sleep and 1 day off between shifts to minimize accumulated fatigue.^1^ Night workers should rest longer than day workers, and the day following a night shift should not be considered part of the recovery time.^1^,^26^

Rotating shifts significantly increase the risk of employee turnover and impact the work quality and safety due to cognitive impairment, which causes lapses in attention and memory, sleep deprivation, mental suffering, lack of empathy, and lower tolerance to stress.^1^,^9^,^27^ Work accidents most often occur during the night shift, after more than 3 consecutive days of work, and when fewer workers on duty.^28^ Furthermore, fatigue due to shift work extends beyond the workplace, since it increases the risk of commuting accidents among workers.^22^

The demands and characteristics of the job must also be considered in the difficult task of mitigating fatigue in shift work. Therefore, physically demanding tasks should not precede monotonous activities or those that require high concentration,^20^ since fatigue-induced reductions in attention and memory and the desynchronization of circadian rhythm negatively affect activities that demand quick reaction time, constant attention, and vigilance.^29^

Fatigue risk must be mitigated to protect the health and safety of shift workers, and strategies to achieve this should be based on a prescribed set of rules, risk management principles, or a combination of both.^1^ These measures should begin with an assessment of worker aptitude and chronotype, so that a work schedule compatible with each worker’s natural sleep and wake times can be established. Maladaptation to shift work increases and precedes a feeling of exhaustion and causes reduced extroversion and social support, while increasing irritability, stress, depression, dependence, and family conflicts.^8^

Fatigue risk management should be based on organizational risks, including indicators of events that may be related to fatigue. This approach should consider the resources and infrastructure available to mitigate these risks and, most importantly, should involve relevant stakeholders, such as workers, their representatives, regulatory agencies, etc.^1^,^30^

Adjusting work schedules (hours worked, shift rotation, rest periods during and between shifts, etc.) is a priority strategy for preventing shift-related fatigue. Other complementary measures help reduce the risk of fatigue, such as the food consumption,^31^ the use of stimulants and new technologies. However, these alternatives should not replace work organization adjustments.^22^,^23^,^32^

Regular consumption of functional foods, especially those containing asparagus extract, helps improve sleep quality, reduces feelings of fatigue, and increases shift work performance.^31^ This simple low-cost strategy can be implemented by companies that provide meals to workers and through workplace health education campaigns.

Caffeine is the most commonly used stimulant and can temporarily improve performance and alertness in people who are sleep deprived.^21^,^22^ Ingesting 200 mg of caffeine 6 hours before starting the night shift has been associated with improved performance.^22^ However, this effect is not observed when caffeine is consumed long before the start of the shift or at the end or after the shift.^21^ Although evidence supports the use of caffeine, it is important to note that it can cause physical symptoms, such as tremors, headaches, and palpitations, and it can interfere with sleep the following day. In addition, constant caffeine use can leads to tolerance, which reduces its benefits compared to more strategic use.^21^,^22^ Thus, the prescription of stimulants or sedatives must be based on a thorough and individualized medical assessment that considers lifestyle habits and work routines to determine the most appropriate strategy.

Blue-light phototherapy is currently being tested to help reduce fatigue from shift work; its powerful effect increases alertness, improving performance and regulating the circadian cycle.^22^,^31^ Providing a calm and comfortable environment that allows for rest and intra-shift naps for 30 minutes between 2:00 a.m. and 4:00 a.m., including the use of blue light therapy glasses, can reduce fatigue and increase the well-being of night shift workers.^31^

Among professional drivers, other effective strategies to mitigate fatigue include installing media systems in vehicles so that workers can listen to music (which helps maintain energy) and fatigue sensors, which emit real-time visual and audio alerts about signs of sleepiness and tiredness.^33^

Despite all these strategies to mitigate and manage shift work-related fatigue, the results tend to be less effective when work schedules infringe on the typical sleep period, i.e., 9:00 p.m. to 9:00 a.m. Thus, a key question when determining shift lengths is whether the risks of continuing to work outweigh the risks of not working.^21^

Although fatigue management is a shared responsibility, employers are primarily responsible, since they must be able to recognize work-related and non-work-related causes, train supervisors to identify fatigue-related behaviors and symptoms in workers, and encourage them to report these symptoms.^1^,^30^ Such education and awareness has proven favorable and results in greater work quality, performance, and safety, in addition to increasing quality of life and reducing stress and risk of burnout.^34^

This study demonstrated the relationship between fatigue and shift work and some of its effects on worker and public health, in addition to presenting updated references from the international literature to help companies implement measures to mitigate and manage fatigue. However, the results should be interpreted with caution in light of possible publication, language, and outcome biases, which are common in this type of research. Furthermore, the studies’ level of evidence was not assessed, which should be considered a study limitation.

## CONCLUSIONS

This review found that the main triggers of fatigue are related to work organization, the demands of work activities, rotating shift characteristics, and rest time. According to the results of the reviewed articles, preventive measures are recommended to minimize workplace fatigue. These measures include: prior assessment of suitability for shift work, chronotype tests, clockwise shift rotations that follow the principles of chronobiology (morning, afternoon, and evening), and limiting work shifts to 5 consecutive days with 11 hours of rest between shifts.

Complementary measures, such as supervised prescription of stimulants (e.g., caffeine) or sedatives for night shift workers with recurring complaints or signs of fatigue, may help manage this problem. In addition, installing equipment to monitor and identify manifestations of fatigue in professional drivers, as well as training workers and supervisors to recognize the signs of physical and mental fatigue, are effective strategies for managing and mitigating fatigue in the workplace.
